# The Effectiveness of Self-Guided Virtual-Reality Exposure Therapy for Public-Speaking Anxiety

**DOI:** 10.3389/fpsyt.2021.694610

**Published:** 2021-08-19

**Authors:** Preethi Premkumar, Nadja Heym, David Joseph Brown, Steven Battersby, Alexander Sumich, Bethany Huntington, Rosie Daly, Eva Zysk

**Affiliations:** ^1^Division of Psychology, London South Bank University, London, United Kingdom; ^2^Department of Psychology, Nottingham Trent University, Nottingham, United Kingdom; ^3^Department of Computer Science, Nottingham Trent University, Nottingham, United Kingdom; ^4^Department of Psychology, University of Nottingham, Nottingham, United Kingdom; ^5^Department of Psychology, University of British Columbia, Vancouver, BC, Canada

**Keywords:** virtual audience, heart rate, negative evaluation, perceived control, social anxiety, head-mounted display

## Abstract

**Objectives:** Self-guided virtual-reality exposure therapy (VRET) is a psychological intervention that enables a person to increase their own exposure to perceived threat. Public-speaking anxiety (PSA) is an anxiety-provoking social situation that is characterized by fear of negative evaluation from an audience. This pilot study aimed to determine whether self-guided VRET (1) increases exposure to PSA-specific virtual social threats, and (2) reduces anxiety, arousal, heartrate and PSA over repeated exposure.

**Methods:** Thirty-two University students (27 completers) with high self-reported public-speaking anxiety attended 2 weekly self-guided VRET sessions. Each session involved the participant delivering a 20-min speech in a virtual classroom. Participants were able to increase their exposure to virtual social threat through the audience size, audience reaction, number of speech prompts, and their own salience in the virtual classroom at 4-min intervals. Participants' heartrates and self-reported anxiety and arousal were monitored during these intervals. Participants completed psychometric assessments after each session and 1 month later.

**Results:** Participants increased their exposure to virtual social threat during each VRET session, which coincided with a reduction in heartrate and self-reported anxiety and arousal. Improvement in PSA occurred post-treatment and 1 month later. The in-session improvement in anxiety correlated with reductions in fear of negative evaluation post-treatment and 1 month later.

**Conclusions:** Increased self-exposure to virtual social threat from self-guided VRET relieves anxiety and shows immediate reductions in subjective and physiological arousal during application, but also yields sustained improvement in PSA.

## Introduction

Social anxiety is, in part, an exaggerated fear of being negatively evaluated by others, for example being criticized, humiliated or rejected during social interaction, observation, and/or in performance situations (1). People with social anxiety disorder (SAD) may appear shy and withdrawn in social situations to mask their immense discomfort and may sometimes avoid social situations altogether (1). SAD has a lifetime prevalence of 4% as per a large multinational epidemiological survey (2). SAD is said to be the third most common psychiatric disorder (3). SAD affects personal relationships, work engagement and academic achievement (4, 5). Yet, SAD is often underdiagnosed (6) and undertreated, with over 80% of people diagnosed with SAD not seeking treatment or having typically lived with their symptoms for 15 to 20 years before seeking treatment (7). Individuals with SAD may not seek treatment for reasons, such as avoidance of face-to-face contact, lack of confidence in treatment, and financial costs (8, 9). Thus, SAD being both highly prevalent and under-treated makes it a large public health concern with psychological and economic costs to the individual and society.

Cognitive-behavioral therapy (CBT), which includes exposure therapy, has become the most evidenced form of intervention for SAD (10, 11). The cognitive element of CBT encourages the patient to question their maladaptive beliefs (10). The exposure element gradually increases the patient's exposure to real (*in vivo*) or imagined social threat. CBT works in a group format and is a natural setting to test social fears with group members (12). Over the last two decades, virtual-reality exposure therapy (VRET) has become a popular digital intervention for various psychological disorders (13, 14). A systematic review of 10 studies showed that VRET was as effective as *in vivo* exposure therapy post-intervention (15). Moreover, a meta-analysis found a large effect size favoring VRET for SAD over waitlist, but a small effect size favoring *in vivo* (i.e., face-to-face) exposure therapy with a therapist over VRET based on six studies (13). *In vivo* exposure therapy may appear to favor VRET for SAD partly because *in vivo* exposure therapy offers a wider range of social situations to rehearse exposure (16). While *in vivo* exposure is effective, many people with social anxiety refuse treatment due to their fear of social situations and the very nature of therapy being a social situation.

VRET is a viable alternative to *in vivo* exposure therapy because patients can encounter social threat in a safe and more predictable virtual environment, and feel that they have control over their exposure to their perceived threat (17). VRET could engage treatment refusers and it has shown efficacy among those who undergo it. VRET may be effective because it could address cognitive biases associated with real social threats, such as having fearful thoughts during public speaking (18) and emotional problems, such as avoidance of and hyperarousal from threat (17). Taken together, VRET offers a promising solution to reduce overall rates of SAD in the population.

Public-speaking anxiety (PSA) is a variant of social anxiety that is frequently encountered by students (19). PSA is a highly anxiety-provoking social situation; it impairs up to 97% of socially anxious individuals (20) and affects 77% of the general population (21). Delivering a public speech in a virtual environment induces as much distress and physiological arousal as delivering a public speech in front of a live audience (22). It significantly increases anxiety and heartrate in socially anxious individuals (22, 23). Research has confirmed that virtual exposure translates to “real life” threat, such as PSA (22). Exposure therapy for social threat often entails delivering a public speech in front of a real or virtual audience (24, 25). VRET can systematically manipulate these social threats, which can induce strong cognitions and high intensity levels of fear (26, 27). These VRET-led improvements in social anxiety are long-lasting and generalize to real world situations (28).

### Self-Guided vs. Therapist-Led VRET for SAD

Therapist-led VRET is where the therapist controls the level of graded virtual exposure according to the patient's hierarchy of fears (16, 24). Self-guided VRET is where the patient controls their own gradual exposure to virtual threat [e.g., (29)]. Self-guided VRET is seen as the latest advance in VRET technology and it produces a meaningful improvement (30). A benefit of self-guided VRET is that it can be easily delivered as homework alongside therapist-led sessions (9). Eight sessions of self-guided VRET for SAD were delivered to individuals with SAD and health controls. These sessions consisted of public-speaking and they produced greater improvement in social anxiety among individuals with SAD than healthy controls (9). Even a single session of self-guided VRET for SAD produced a large improvement in PSA in individuals self-reporting high PSA (31). Two studies on acrophobia (fear of heights) found that symptoms of acrophobia improved to a greater extent (with large effect sizes) when receiving six modules of VR-CBT from a virtual therapist over 2 or 3 weeks compared to the wait-list group (32, 33). One likely reason for the efficacy of self-guided VRET is perceived control. According to the Health Belief Model, patients are more likely to engage in and comply with therapy if they believe to have control over treatment (34, 35). Such perceived control could denote resilience to social stress (36), decision-making (37) and cognitive reappraisal (38). Therapist-led VRET focuses more on establishing a good therapeutic alliance through agreement on therapeutic tasks and goals to achieve visible treatment outcomes, such as treatment adherence (39). Therapist-led VRET focuses less on supporting the client toward gaining autonomy (39) and control over exposure without risk of over-exposure to threat (40). Having a higher level of perceived control over exposure to threat encourages the person to approach the threat, at least among individuals with arachnophobia (40).

Self-guided VRET could enable such autonomy and control. According to the perceptual control theory (41), control involves keeping a perceptual variable (e.g., perceived distance from a threat) at a selected state through comparing its current value with a reference value that drives actions to counteract disturbances to that variable. 'Perceived' control could be defined as the consciously reportable experience of the amount of control over a specific variable (e.g., the verbal report of amount of control over perceived distance from a threat). Self-guided VRET could enhance control through providing a hierarchy of virtual threats and allowing the client to select the steps needed to reach a goal through graded exposure, for example, gradually reducing the distance from the audience. Future studies of self-guided VRET should assess client control within the virtual environment and how it affects the effectiveness of the intervention.

Manipulating certain elements of virtual social threat during self-guided exposure could improve the efficacy of self-guided VRET (42, 43). These social elements could include (1) the audience size (24), (2) the reaction of the avatar audience (26), (3) the proximity to the audience (31), (4) the number of speech prompts available for delivering a speech (44), and (5) the salience or presence of the self in the virtual classroom (45). Manipulating the audience size is well-documented to increase exposure within VRET for SAD [c.f. (24)]. In contrast, the reaction of the audience has been manipulated less often, with studies often defaulting to a neutral audience reaction (44). Manipulating the audience reaction is crucial for addressing the fear of human evaluation, whether positive or negative, as the fear of human evaluation is a core fear of social anxiety (46, 47). Fear of negative evaluation predicts response to treatment for SAD (48). Importantly, negative reactions from the virtual audience have been found to evoke social anxiety in spite of participants being aware that the members of the audience are merely fictitious (26). The proximity to the audience is another factor to be considered for manipulation, as this manipulation could alter the attention of the participants to the audience. Being closer to the audience could encourage the socially anxious person to focus on the audience rather than themselves, thus improving eye-contact and fluency (49). The speaker's close proximity to the audience, especially among individuals with PSA, could mimic the feeling of their performance being closely scrutinized (50). Thus, gradual exposure could help to overcome this sense of scrutiny. Salience of the self in the virtual classroom is another factor that could gauge the speaker's awareness of being in the virtual space and increase their sense of presence. Creating a sense of presence in the virtual environment is important. Presence is the participant's psychological response to a virtual environment (45) in terms of their sense of immersion and emotions, such as anxiety (51, 52). A head-mounted display of virtual social interactions increases presence more than a screen-projected display does (53).

Measuring physiological arousal during VRET would objectively measure speech and performance anxiety. Delivering a speech in front of a virtual audience increases anxiety and heartrate in socially anxious individuals (22, 23). In contrast, patients with SAD have a lower heartrate than people with moderate social anxiety while monitoring their own performance when under public scrutiny (54); this finding could suggest a breakdown of the physiological stress response system due to performance anxiety. The physiological stress response is compromised in clinical social anxiety; yet, a 4-week therapist-guided VRET for PSA reduces heartrate (55). Thus, physiological arousal could be an objective measure of the psychological response to VRET.

The current pilot study aimed to test the feasibility of self-guided VRET for PSA in a sub-clinical group of university students who self-reported high PSA. It was hypothesized that (1) participants would gradually increase their exposure at their own pace to the five aforementioned elements of social threat during the self-guided VRET; (2) the gradual exposure to social threat would produce a concomitant reduction in anxiety, arousal and heartrate within the virtual environment; (3) self-guided VRET would reduce PSA at post-intervention and 1-month follow-up timepoints, and (4) changes in anxiety, arousal and heartrate during the VRET sessions would relate to improvement in PSA at post*-*intervention and 1-month follow-up timepoints.

## Materials and Methods

### Participants

Thirty-two participants were invited to take part in the experiment on the basis of scoring the highest on the Speech Anxiety Thoughts Inventory (SATI) (56) among a large participant pool of 336 students. These 336 students were recruited for potential inclusion in this social anxiety study if they met the inclusion criteria and had completed the SATI in an online survey among other several self-report measures (see section Materials and Assessments). The 32 participants greatly surpassed the inclusion criterion of scoring 1.5 SD above the mean SATI score [mean (SD) = 54.34 (18.35)] in an independent normative sample (*n* = 548) (56). The mean (SD, range) SATI score = 96.7 (7.8, 82–111) of the 32 participants was 2.3 SD above the mean of the normative sample (56) and one SD above the mean of the current screening survey sample (*n* = 336). Further inclusion criteria were being aged 18+ years, a university student, able to speak English fluently and having normal or corrected vision with contact lenses. Participants' ages ranged from 18 to 40 years (mean = 21.4, SD = 4.9) and mostly identified as female (*n* = 27, 84.4%) (see Table 1). All participants were psychology students (28 undergraduates, 4 postgraduates). Twenty-seven (84.4%) were Caucasian, three were African-Caribbean, one was Asian and one was mixed race. English was either their first language (87.5%) or second language (12.5%). Participants ranged from never having been diagnosed with SAD (84%) to having a current diagnosis (6.0%) or a past diagnosis of SAD (6.0%); one participant chose not to declare their diagnostic status. Individuals who were currently engaging in SAD psychotherapy were excluded.

**Table 1:** Demographic characteristics and social anxiety of completers (*n* = 21) and non-completers (*n* = 11).

**Characteristic**	**Completers**	**Non-completers**	**T statistic or chi-square (*df*)**	***P*-value**	**Effect size (η^*2*^)**
*N*	21	11			
Age, mean (S.D.)	21.57 (5.00)	21.00 (4.98)	0.3 (30)	0.760	0.11
Gender, % female	76.2	100	3.1	0.08	
Ethnicity, % White	90.5	72.5	1.7	0.19	
SAD diagnosis, %	9.5	18.2	0.5	0.48	
**Social anxiety at baseline**					
SATI	97.71 (7.44)	94.64 (8.35)	1.07 (30)	0.295	0.40
PSAS^*^	4.26 (0.31)	4.3 (0.66)	0.39 (30)	0.693	0.15
PRCS^*^	1.17 (0.10)	1.19 (0.20)	0.43 (30)	0.669	0.16
BFNE^*^	50.67 (7.14)	47.81 (13.62)	0.78 (30)	0.440	0.29
LSPS—*P-anx*	20.29 (6.10)	21.87 (6.9)	0.65 (30)	0.523	0.24
LSPS—*P-avoid*	18.62 (6.14)	16.54 (7.53)	0.84 (30)	0.408	0.31
LSPS—*S-anx*	18.57 (8.18)	18.91 (6.95)	0.12 (30)	0.908	0.04
LSPS—*S-avoid*	17.00 (7.79)	15.91 (7.27)	0.38 (30)	0.703	0.14
SUDS avoidance	85.71 (22.26)	97.27 (2.47)	1.67 (30)	0.105	0.62
**Social anxiety at session one**					
SATI^*^	87.86 (15.05)	79.00 (24.71)	1.26 (30)	0.290	0.47
PSA	3.87 (0.59)	3.57 (0.75)	1.22 (30)	0.234	0.45
PRCS-SF	1.25 (0.16)	1.32 (0.28)	0.82 (30)	0.418	0.31
SUDS avoidance	56.67 (20.33)	53.64 (25.80)	0.36 (30)	0.718	0.14

* *Homogeneity of variance not assumed, but uncorrected degrees of freedom are reported; BFNE, brief fear of negative evaluation; LSAS—Liebowitz Social Anxiety Scale: subscales: P-anx, performance anxiety; P-avoid, performance avoidance; S-anx, social anxiety; S-avoid, Social avoidance; PSAS, public speaking anxiety scale; PRCS-SF, personal report of confidence as a speaker—short form; SATI, social anxiety thoughts inventory*.

### Assessments

#### Speech Anxiety Thoughts Inventory

This 23-item scale assesses negative thoughts related to speech anxiety, such as “I worry that I will be asked to give a speech.” Items are rated from 1 (“I do not believe the statement at all”) to 5 (“I completely believe the statement”). The overall score was the total of individual items. The scale has two factors, namely ‘prediction of poor performance' and ‘fear of negative evaluation by audience'. The mean (SD) of the SATI has been previously reported to be 54.34 (SD = 18.35) in Psychology undergraduate students (*n* = 547) (56). The scale has convergent validity with other measures of public speaking (56). Internal consistency was good in the current sample (Cronbach's α = 0.80).

#### Public Speaking Anxiety Scale

The PSAS assesses the manifestation of cognitive, behavioral and physiological responses to PSA. It contains 17 items, such as “Giving a speech is terrifying.” Each item is scored from 1 (“Not at all”) to 5 (“Extremely”), with 5 items being reverse-coded. The mean score of individual items is calculated. The scale has demonstrated concurrent, convergent and discriminant validity, and high internal consistency (Cronbach's α = 0.94) in a previous study (57), and good internal consistency (Cronbach's α = 0.85) in the current study.

#### Personal Report of Confidence as a Speaker—Short Form

The PRCS-SF is a 12-item scale that assesses behavioral responses, such as “My posture feels strained and unnatural.” It assesses affective responses to public-speaking situations, such as “I am fearful and tense all the while I am speaking before a group of people.” Participants answer “True” = 1 or “False” = 2 for each item. The overall score was calculated as the mean of individual items, so that the overall score ranged from 1 to 2, with a higher score indicating more confidence as a speaker. The PRCS-SF had good internal consistency in a previous study (Cronbach's α = 0.85) (58), but was weaker in the current study (Cronbach's α = 0.60). The PRCS-SF has good convergent validity as determined by its relationship with measures of public-speaking ability (59).

#### Liebowitz Social Anxiety Scale

This 24-item scale (60) assesses fear and avoidance of social interaction situations, such as attending a party and meeting strangers, and performance situations, such as eating in public and taking a test. Each situation is assessed from 0 (none) to 4 (severe) on fear, and from 0 (never: 0%) to 3 (usually: 67–100%) on frequency of avoidance. The overall score and subscale scores are the totals of individual items. The scale has four subscales with the following means (SD) in a normative sample of 382 patients with SAD (61): Fear of Social Interaction = 16.9 (7.7); Avoidance of Social Interaction = 15.7 (8.2); Fear of Performance = 18.6 (6.8); and Avoidance of Performance = 16.0 (7.3). The scale has shown convergent validity with other measures of social phobia and good internal consistency (Cronbach's α = 0.96) in a previous study (61) and the current study, α = 0.96.

#### Brief Fear of Negative Evaluation Revised Scale

This 12-item measure of fear of negative evaluation includes items, such as “I am frequently afraid of other people noticing my shortcomings.” Items are rated from 0 (“Not at all characteristic of me”) to 4 (“Extremely characteristic of me”). The overall score is the total score of individual items after reverse-coding positively-worded items. The mean (SD) of the BFNE in a sample of 201 undergraduate students was 30.7 (9.04) (62). The scale has shown discriminant and convergent validity and good internal consistency (Cronbach's α = 0.97) in a previous study (62) and the current sample.

### Subjective Units of Distress Scale

The SUDS (63) is a visual analog scale that reliably measures subjective fear (64). It is sensitive to change in mental state (65). The SUDSs for anxiety and arousal were integrated and administered directly in the virtual environment through a scale ranging from “Not at all” (0) to “Extremely” (100). The anxiety and arousal questions were “How anxious do you feel right now?” and “How aroused do you feel right now?” Anxiety was defined as dryness of mouth, difficulty breathing, trembling, feeling panicked and increased heart rate (66). Arousal was defined as feeling active, vigorous, lively, energetic and alert, and not tired, sleepy, drowsy, or passive (67). The behavioral avoidance question was “How much do you wish to avoid giving another speech?,” and it was administered before and after each VR session along with the other self-report scales, where participants responded on a 0–10 Likert scale from “Not at all” to “Very much.”

### Heart Rate

Heartrate was measured from Microsoft Band 2, a biometric wristband, during the four 1-min intervals following each speech block over the 20-min VRET-led speech. Heartrate was sampled every 4 s. The average heartrate was calculated in beats per minute during each of the four intervals.

### Virtual-Reality Exposure Therapy

#### Software and Hardware

The VRET was developed using the Unity real-time 3D development platform (68). The Unity-based VRET smartphone application was deployed to the Android operating system. Data on heartrate were collected through the smartphone application, since the VRET smartphone application was connected to the Microsoft Band 2, a biometric wristband. A bespoke plugin developed in Java acted as a bridge between the Java-based official Microsoft Band software development kit and the VRET smartphone application. A Samsung Gear VR headset housed a Samsung Galaxy S7 smartphone on which the VRET application ran to display the virtual environment.

#### Virtual Environment Design and Self-Guided Manipulation

**Figure 1:**
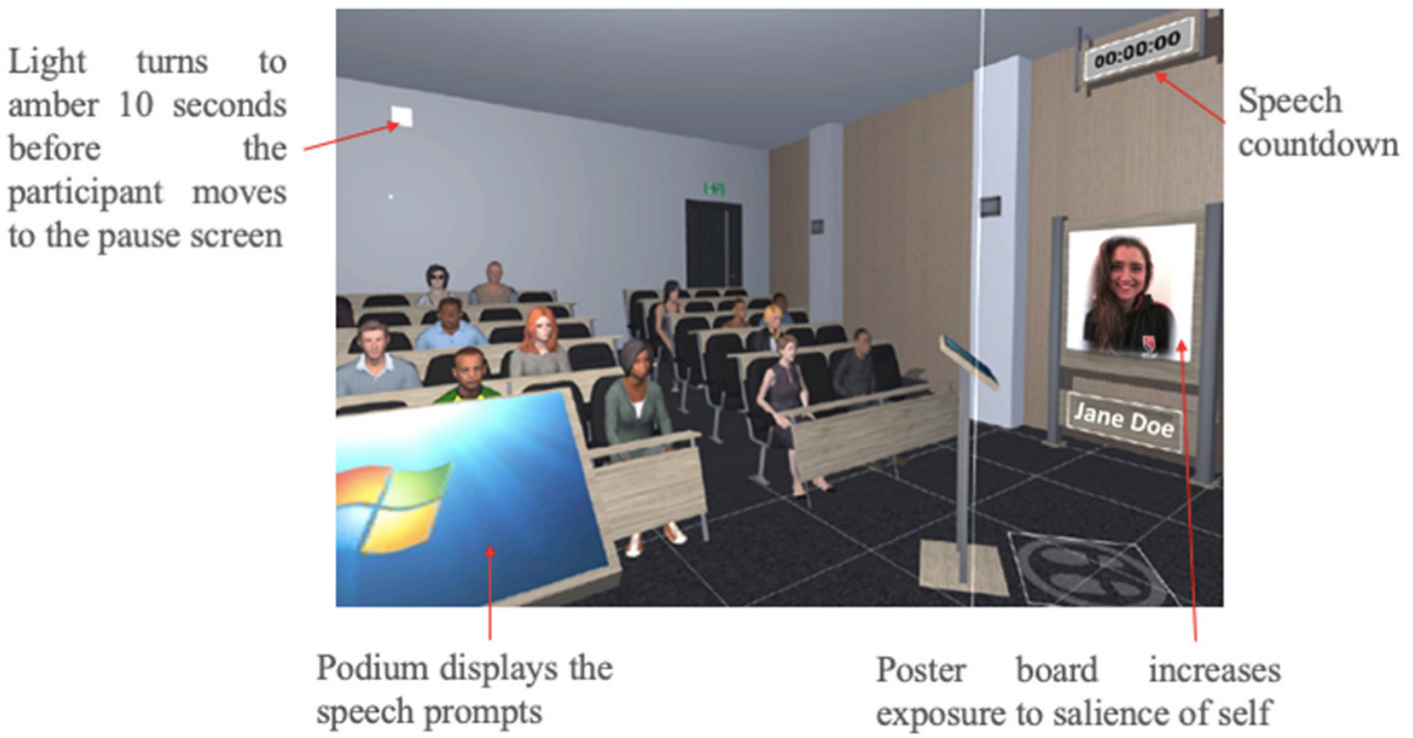
Display of the features of the virtual classroom.

Participants gave a 20-min speech in a virtual classroom on the topic of “the experience of being a university student” following a previous study (45). The speech was broken into four 5-min blocks. Participants spoke spontaneously by following prompts that appeared in the virtual environment. The prompts included general knowledge about the University and its facilities, impressions about the course, level of academic support, extracurricular activities and social activities. Participants were encouraged to increase their exposure to the virtual social threat at their own pace. After every 5-min speech block, participants had a brief (1 minute) interval when they entered a virtual pause menu. Here, participants could respond to the SUDS on anxiety and arousal and navigate to a settings menu where they could manipulate the five elements of social threat (Figure 1). Each modifiable element had three grades (G) of exposure, from low, moderate to high level of exposure: (i) audience size—six (G1), 12 (G2) or 20 (G3) people; (ii) audience reaction—approving (G1), neutral (G2) or disapproving (G3); (iii) speaker's distance from the audience—far (G1), near (G2) or nearest (G3); (iv) number of speech prompts per slide—many (G1), moderate (G2) or few (G3); and (v) salience of self—no poster (G1), a silhouette with the label “Speaker” (G2), or a photo of the participant and their full name (G3). The speech prompts (with suggested points to speak about) appeared on the virtual podium as bullet points on PowerPoint slides through which the participant could scroll using the controls on the Samsung Gear VR headset. All participants were started on Grade 1 of each element of the VRET settings at Session 1. A countdown appeared inside the virtual classroom to allow participants to track the remaining time of their speech. Participants were given a 10-s warning by means of a signal turning from white to amber in the virtual lecture room before they were taken to the pause menu.

Due to a programming error, the podium disappeared when the participants changed their position from the default position to a different position; however, most participants chose not to manipulate the distance from the audience. Hence, the analyses excluded the data on the manipulation of distance from audience.

### Procedure

**Figure 2:**
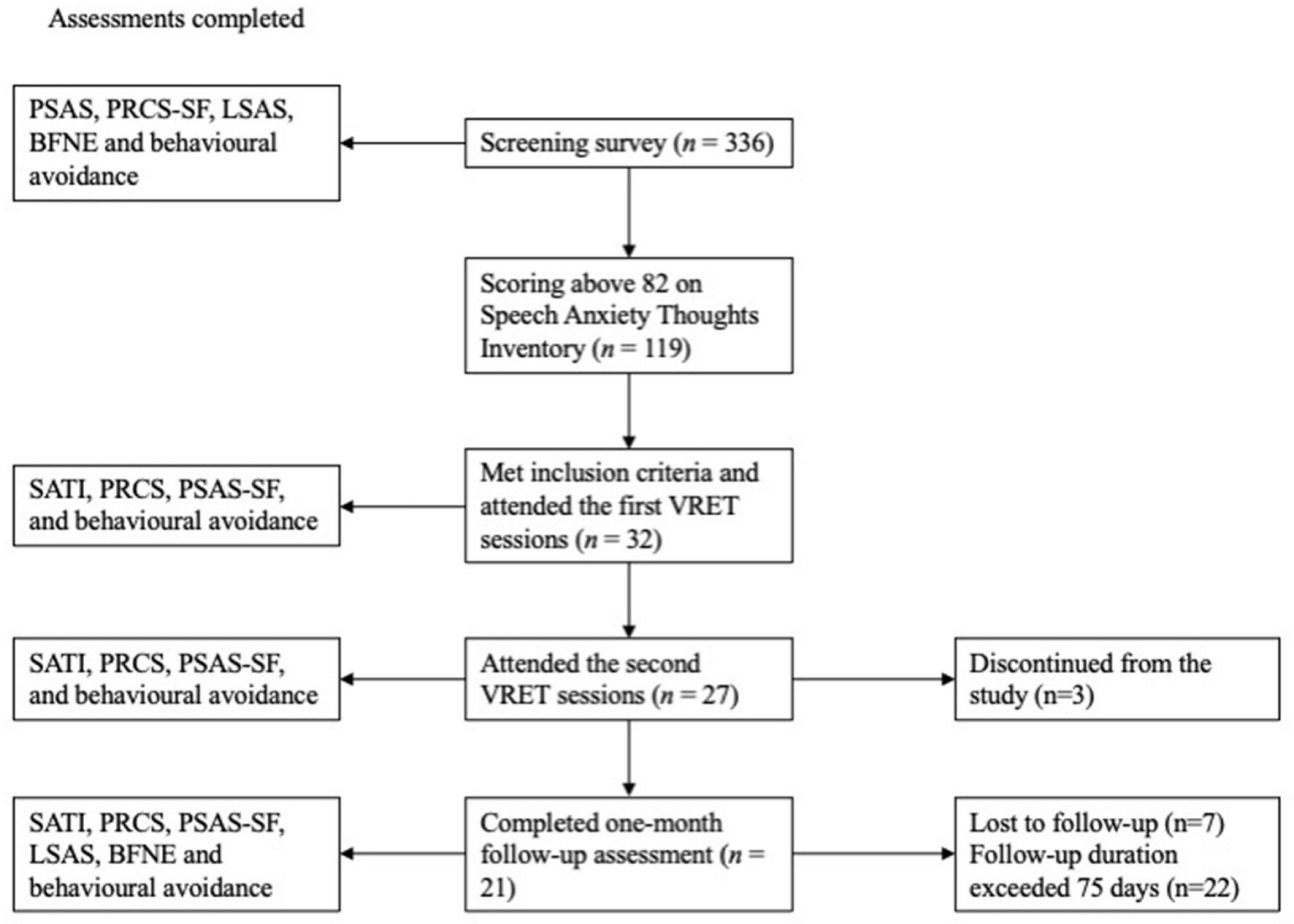
Flow diagram of participant retention at each stage of the study; BFNE, brief fear of negative evaluation scale; LSAS, Liebowitz social anxiety scale; PRCS-SF, personal report of confidence as a speaker; PSAS-SF, Public-speaking anxiety scale—short form; SATI, speech anxiety thoughts inventory; SUDS, subjective units of distress scale.

Invited participants completed the online screening survey on an average of 60 days (median = 46 days, SD = 59.4) before they took part in Session 1. The screening survey comprised the SATI, PSAS, PRCS-SF, LSAS, BFNE and SUDS for behavioral avoidance (Figure 2). Participants who fulfilled the selection criteria for the highest SATI scores were invited to attend the 2 weekly hour-long sessions (number of days between sessions mean = 7.8, median = 7, SD = 5.3). Participants were given a hard copy of the PowerPoint slides containing the speech prompts a few minutes before they wore the VR headset to familiarize themselves with the suggested speaking points. Participants were given the following instructions,

‘*You will have three minutes to look over the notes before we begin the virtual-reality experiment. You will see the notes in the VR environment. Don't read the notes – talk about what you want to talk about regarding your experiences. The notes are there to give you prompts when you run out of things to say. Don't worry if you go “off topic”! The aim is to keep you talking for 20 minutes, and NOT the quality of your presentation. Make it personal – give your views and opinions, and share personal stories and examples. Don't rush. Speak slowly and clearly. Spend time elaborating on the notes. You can switch to a higher level on any of the features I mentioned about whenever you enter the pause menu. You are encouraged to switch to a higher level in any of these individual areas whenever you feel comfortable.'*

Participants engaged in the 20-min VRET speech in 5-min blocks, which was interspersed by four up-to-1-min intervals to allow the participant to manipulate the environment, should they choose to. Participants completed the self-report questionnaires at the end of each 20-min session and 1 month after the second session (number of days between Session 2 and follow-up mean = 56.9, median = 45, SD = 42.5). The Business, Law and Social Sciences College Research Ethics Committee at Nottingham Trent University approved the study (ethics application number No. 2017/82). Participants gave informed consent and were given a £10 shopping voucher for each experimental session attended and awarded research credits.

### Statistical Analyses

Thirty-two participants completed Session 1, 27 completed Session 2, and 21 completed the follow-up assessment (Figure 2; note that data from two participants exceeded the 75-day follow-up limit and were excluded at follow-up). Participants were informed that they could withdraw without giving a reason. Final completers (*n* = 21) and non-completers (*n* = 11) did not differ demographically or on any self-report measure at baseline or at the end of Session 1 (Table 1). Multiple imputation was used to replace the missing values of the self-report assessments and heartrate during the VRET sessions [*c.f*. (69)]. An iterative Markov chain Monte Carlo (MCMC) method was used to perform the multiple imputation due to the monotonic nature of the missing responses. Data on the levels of exposure to each element that participants could manipulate were missing, but not replaced due to their ordinal nature.

A separate analysis of variance (ANOVA) was performed on each VRET session with time as the independent variable (four pauses) and the four elements of graded exposure as the dependent variables (hypothesis 1). Further ANOVAs were performed with time (×5 for anxiety and arousal SUDS and ×6 for heartrate) and session (×2) as independent variables, and anxiety SUDS, arousal SUDS and heartrate as the dependent variables (hypothesis 2). An ANOVA was performed with time (×3, baseline, post-treatment and 1-month follow-up) as the independent variable and the scores on SATI, PSAS, PRCS-SF, avoidance of giving a speech, BFNE and LSAS—*fear of performance* as the dependent variables (hypothesis 3). *Post hoc* Bonferroni-corrected pairwise comparisons compared timepoints. The change in anxiety, relative to baseline, was calculated as: (anxiety at baseline – anxiety post Session 2 or at follow-up)/anxiety at baseline. The change, relative to baseline, in SUDS anxiety and arousal was correlated against the change, relative to baseline, in SATI, PRCS-SF, PSAS, LSAS and BFNE post-treatment and at 1-month follow-up (hypothesis 4).

## Results

### Graded Exposure to Social Threat in the Virtual Environment

**Figure 3:**
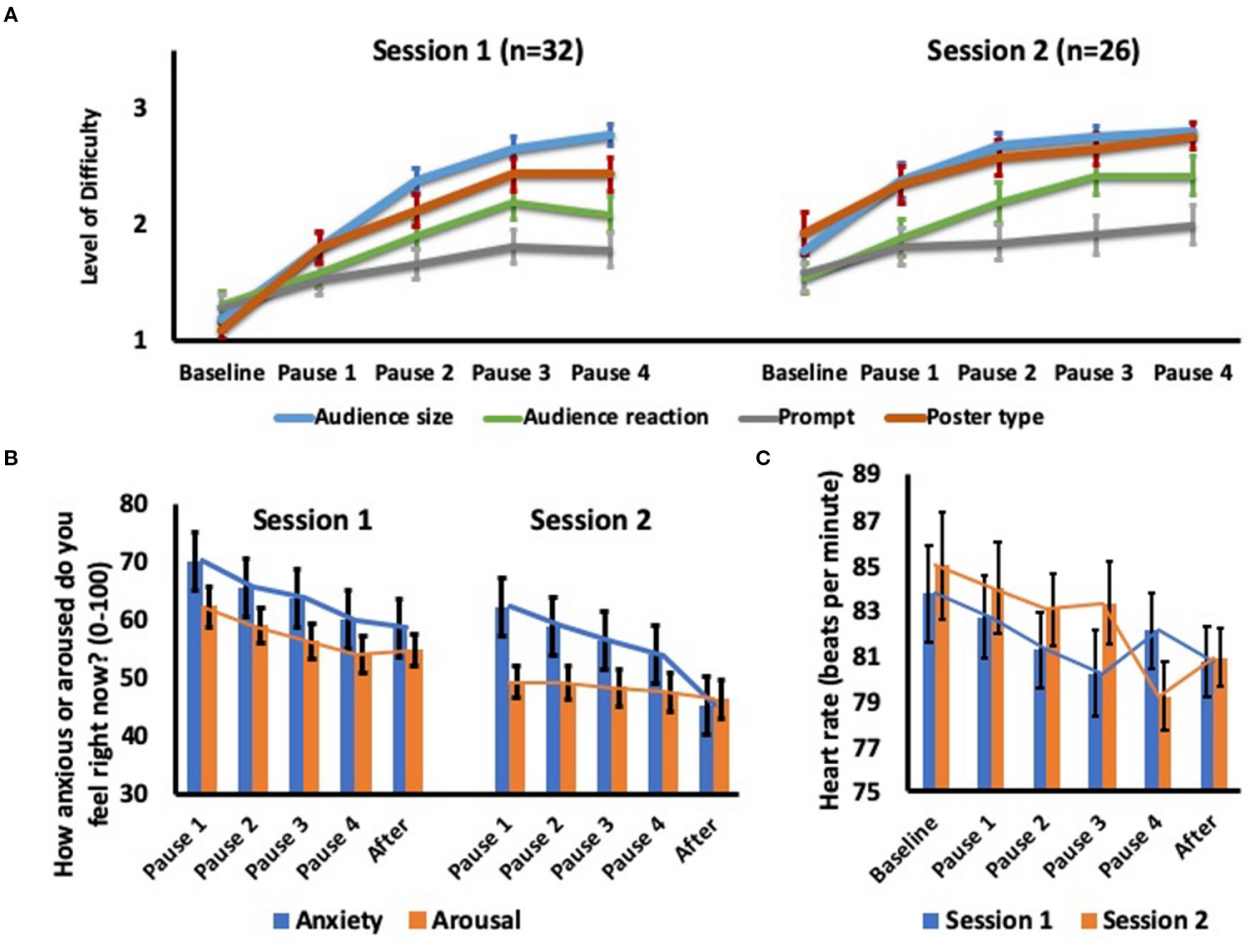
Participant changes in exposure to social threat at each 4-min pause within the virtual environment in **(A)** modifying the elements of the social threat, **(B)** anxiety and arousal and **(C)** heartrate.

Participants chose to increase their self-guided exposure to audience size, audience reaction and salience of self by the last pause of Session 1 relative to the first pause of Session 1 (Table 2 and Figure 3A). Likewise, participants chose to increase their self-guided exposure by the last pause of Session 2 relative to the first pause of Session 2. The level of the number of speech prompts did not change significantly in either session. Participants also exhibited greater exposure to audience size, *F*_(1,26)_ = 43.87, *p* < 0.001, η^*2*^ = 0.63; audience reaction, *F*_(1,26)_ = 10.98, *p* = 0.003, η^*2*^ = 0.30; number of prompts, *F*_(1,26)_ = 4.97, *p* = 0.035, η^*2*^ = 0.16; and salience of self, *F*_(1,26)_ = 26.08, *p* < 0.001, η^*2*^ = 0.50, at the last pause of Session 2 relative to the first pause of Session 1.

**Table 2:** Self-guided exposure to social threat within the virtual environment.

	**Pause 1**	**Pause 2**	**Pause 3**	**Pause 4**	***F*-statistic (df)**	***P*-value**	**Effect size (η^*2*^)**
**Session 1 (** ***n*** **= 32)**							
Audience size	1.81 (0.64)	2.38 (0.61)	2.66 (0.54)	2.78 (0.49)	30.36 (3, 93)	<0.001	0.49
Audience reaction	1.59 (0.76)	1.91 (0.69)	2.19 (0.82)	2.09 (0.86)	4.62 (3, 93)	0.005	0.13
Number of prompts	1.53 (0.72)	1.66 (0.74)	1.81 (0.82)	1.78 (0.83)	2.17 (3, 93)	0.121	0.06
Salience of self	1.81 (0.78)	2.12 (0.79)	2.44 (0.80)	2.44 (0.84)	12.44 (3, 93)	<0.001	0.29
**Session 2 (** ***n*** **= 25)**							
Audience size	2.37 (0.74)	2.67 (0.55)	2.74 (0.45)	2.81 (0.40)	7.31 (3, 78)	0.002	0.22
Audience reaction	1.85 (0.82)	2.18 (0.88)	2.41 (0.84)	2.41 (0.84)	5.88 (3, 78)	0.007	0.18
Number of prompts	1.78 (0.80)	1.81 (0.79)	1.93 (0.83)	2.00 (0.83)	2.10 (3, 78)	0.143	0.07
Salience of self	2.30 (0.82)	2.55 (0.75)	2.66 (0.68)	2.78 (0.58)	6.83 (3, 78)	0.002	0.21

*The podium did not appear when the participant moved to a higher level of threat exposure due to a programming error; most participants choose not to manipulate this element, so results for manipulation of distance from audience are not reported*.

### Changes in Anxiety, Arousal and Heartrate During the VRET Sessions

There was a main effect of time on SUDS-anxiety over the two sessions *F*_(4,124)_ = 9.24, *p* < 0.001, η^*2*^ = 0.23 (Figure 3B). *Post hoc* Bonferroni-corrected pairwise comparisons revealed reduced anxiety by the end of each VRET session relative to the first two pauses, *p* ≤ 0.001. There was a main effect of session on SUDS-anxiety, *F*_(1,31)_ = 30.77, *p* < 0.001, η^*2*^ = 0.50. SUDS-anxiety decreased by Session 2 relative to the first pause of Session 1, mean difference = 24.94, *F*_(1,31)_ = 40.33, *p* < 0.001, η^*2*^ = 0.56.

There was a significant main effect of session, *F*_(1,31)_ = 11.87, *p* = 0.002, η^*2*^ = 0.28. There was no main effect of time on SUDS-arousal, *F*_(4,124)_ = 2.60, *p* = 0.08, η^*2*^ = 0.08. Arousal was lower at Session 2 than at session 1. SUDS-arousal decreased by Session 2 relative to baseline, mean difference = 15.99, *F*_(1,31)_ = 10.02, *p* = 0.003, η^*2*^ = 0.24. There was a main effect of time on heartrate, *F*_(5,155)_ = 3.00, *p* = 0.013, η^*2*^ = 0.09, but no main effect of session on heartrate, *F*_(1,31)_ = 0.30, *p* = 0.59, η^*2*^ = 0.01 (Figure 3C). Heartrate decreased by the end of Session 2 relative to baseline, mean difference = 4.55, SD = 11.01, *F*_(1,31)_ = 5.48, *p* = 0.002, η^*2*^ = 0.15.

### Change in Self-Reported PSA Over Time

There was a significant main effect of time on PSA as measured by SATI, PSAS, PRCS-SF, avoidance of giving a speech (single item question), BFNE and LSAS –* fear of performance* (Table 3). Bonferroni-corrected *post hoc* pairwise comparisons revealed improvement at Session 1, Session 2 and 1-month follow-up relative to baseline, *p* ≤ 0.01, on the SATI, PSAS and avoidance of giving a speech. PSAS and PRCS-SF scores improved at Session 2 relative to Session 1, *p* ≤ 0.01. BFNE and LSAS—*fear of performance* scores improved at follow-up relative to baseline and Session 2, *p* < 0.02. Only the SATI score improved at follow-up relative to both Sessions 1 and 2, *p* < 0.03. PRCS-SF scores declined at follow-up relative to Session 2, *p* < 0.001.

**Table 3:** Change in PSA from baseline, to Session 1, Session 2 and one-month follow-up.

**Measure**	**Baseline (A)**	**Session 1 (B)**	**Session 2 (C)**	**1-month follow-up (D)**	***F* (df)**	***P-*value**	**Effect size (η^*2*^)**	**Pairwise comparisons**
SATI	96.65 (7.77)	84.81 (19.00)	78.84 (20.06)	71.18 (17.99)	21.80 (3, 93)	<0.001	0.41	A>B^*^, A>C and D^***^, B>D^**^, C>D^*^
PSAS	4.29 (0.45)	3.76 (0.65)	3.42 (0.73)	3.54 (0.69)	18.9 (3, 93)	<0.001	0.38	A>B, C and D^***^, B>C^**^
PRCS-SF	1.17 (0.14)	1.28 (0.21)	2.15 (0.24)	1.36 (0.22)	214.1 (3, 93)	<0.001	0.87	A < C and D^***^, B < C^***^, D < C^***^
Speech avoidance	89.69 (19.10)	55.62 (21.99)	53.77 (23.59)	47.48 (20.82)	38.19 (3, 93)	<0.001	0.55	A>B, C and D^***^
BFNE	49.68 (9.73)	-	46.70 (9.59)	43.10 (9.31)	8.93 (2, 62)	0.002	0.22	A>D^**^, C>D^*^
LSAS—*P-anx*	20.81 (6.31)	-	20.02 (6.13)	17.57 (6.64)	5.67 (2, 62)	0.005	0.16	A>D^*^, C>D^*^
LSAS—*P-avoid*	17.91 (6.60)	-	17.31 (6.22)	15.82 (5.49)	2.03 (2, 62)	0.140	0.06	
LSAS—*S-anx*	18.69 (7.66)	-	17.69 (6.66)	16.44 (7.01)	2.48 (2, 62)	0.092	0.07	
LSAS—*S-avoid*	16.62 (7.52)	-	16.17 (6.39)	14.59 (6.18)	1.64 (2, 62)	0.203	0.05	

* *p < 0.05*;

** *p < 0.01*;

*** *p < 0.001; BFNE, brief fear of negative evaluation; LSAS, Liebowitz social anxiety scale; LSAS subscales: P-anx, performance anxiety; P-avoid, performance avoidance; S-anx, social anxiety; S-avoid, social avoidance; PRCS-SF, personal report of confidence as a speaker—short form; PSAS, public speaking anxiety scale; SATI, social anxiety thoughts inventory*.

### Correlation Between Change in Anxiety and Arousal During VRET Sessions With Change in PSA

Improvement in SUDS-anxiety from the first pause of Session 1 to post-Session 2 correlated with (1) improvement in PSAS pre-therapy to post-Session 2, *r* = 0.40, *p* = 0.023, (2) improvement in BFNE 2 pre-therapy to post-Session 2, *r* = 0.40, *p* = 0.022, and (3) improvement in BFNE pre-therapy to follow-up, *r* = 0.44, *p* = 0.012.

## Discussion

This is the first study to systematically examine the feasibility of self-guided VRET for PSA. This self-guided VRET aims to encourage individuals with high self-reported PSA to voluntarily pace their gradual exposure to virtual social threat (hypothesis 1). These findings support the hypotheses that reductions in self-reported anxiety and physiological arousal can accompany the ongoing self-guided desensitization to virtual social threat (hypothesis 2). Furthermore, self-guided VRET improves PSA after intervention and at 1-month follow-up (hypothesis 3). Finally, a reduction in anxiety during the VRET sessions relates to an overall improvement in PSA after the intervention and 1 month later (hypothesis 4). These findings are discussed further.

On average, participants increased their exposure to all four available elements of social threat over the course of the two VRET sessions. Within each session, participants (on average) increased their graded exposure to three out of the four elements of social threat, namely audience size, audience reaction and salience of self, and participants made full use of the range of exposures offered. This preliminary evidence suggests that self-guided exposure has the potential to desensitize individuals with high PSA to social threat without risking exposure to excessive fear. The possible health beliefs that accompany this improvement could be that participants gain a sense of control over one's health and feel empowered and motivated to engage with treatment (34, 35). Future studies could explicitly test the role of health beliefs when engaging in self-guided VRET.

Alongside this increased exposure to virtual social threat, the self-guided VRET produced reductions in anxiety during the VRET sessions, improved subjective and physiological levels of arousal (heartrate). Additionally, participants showed overall improvement in PSA across the two sessions. These findings suggest that self-pacing one's exposure to virtual social threat could reliably alleviate anxiety and arousal when using the application. In addition, the VRET-linked reduction in anxiety found during the VRET sessions related to an overall improvement in PSA after the two sessions and to a further improvement in fear of negative evaluation 1 month later. Hence, these improvements could be linked to long-term improvement in fear of negative evaluation. Exposure to social threats within the virtual environment could mean reduced perceived social anxiety in real life, such as being concerned about social judgment. Less anxiety within the virtual environment does translate to less anxiety in real life, since VRET reduces real-life self-reported anxiety and length of speech during a speech in front of an audience (24, 25). The self-paced exposure to virtual social threat could encourage effortful emotion regulation (70). The relief in anxiety during application could modify cognitive elements of PSA, such as reevaluation of irrational beliefs, anticipated anxious rumination and self-referential bias (71, 72). Following the intervention, a participant informed the research team: ‘*I did a presentation last week. While I was still anxious and I found my heart pounded, I definitely noticed a difference! I didn't stutter and I was able to look my audience in the eyes. I'm definitely still anxious with presentations, but it's made me more able to face them*.' Again, future investigations should examine such mechanisms of emotion regulation and perceived control that aid improvement in fear of negative evaluation.

The maintenance of the improvement in PSA 1 month later could suggest that self-guided VRET addresses the core features of PSA, namely fear of negative evaluation and fear of performance. Fear of negative evaluation is a key feature of social anxiety. It is characterized by a strong negative self-referential bias and irrational thoughts, such as worrying about how others feel about you and perceiving criticism and rejection from others (73). The self-guided VRET may help clients to challenge their beliefs and biases toward the virtual social threats, such as virtual audience members shaking their heads, and to transfer these skills to real life. Virtual exposure to threat-provoking situations, including public-speaking, translates to “real life” threat (22). This improvement in fear of negative evaluation following VRET is consistent with the findings of Anderson et al.'s (24) study, but not Kampmann et al.'s (16) study. Participants who received therapist-led VRET and performed homework assignments alongside the VRET showed an improvement in fear of negative evaluation (24). Participants who did not perform homework assignments did not show this improvement (16). The self-guided VRET might challenge perceptions of social threat in real life. Setting homework assignments for socially anxious individuals to practice these skills could have added long-term value following self-guided VRET. Future investigations should determine how long the improvement in PSA is sustained. For example, it is known that a single session of self-guided VRET for fear of spiders can sustain reduced anxiety for up to 12 months post-treatment (43), and self-guided VRET for SAD may offer similar effects.

### The Psychophysiological Mechanisms of Responsiveness to Self-Guided VRET

Physiological habituation happens when adapting to stress. High social anxiety can delay this habituation (74). The current study found a reduction in heartrate of 4.5 beats per minute by the end of VRET Session 2 relative to baseline, and this reduction equated to large effect size. This reduction in heartrate suggests habituation to delivering a speech to the virtual audience. A virtual exposure to social threat over a 4-week period as part of a therapist-guided VRET for PSA has previously shown to reduce heartrate (55). In contrast, other research has shown that brief, 3-minute, exposure to virtual social threat does not change heartrate when the virtual audience gradually increases its display of threat (23). Thus, the duration of exposure to social threat may determine the amount of physiological habituation.

### Limitations, Technological Advances to Enhance the VRET Experience and Therapeutic Implications

This study was a feasibility study. It did not include a control intervention, such as a virtual-reality-guided breathing exercise, and so it did not determine whether a routine 20-min exercise would produce a similar improvement in PSA, as participants naturally regress to the mean. A full randomized-controlled trial must test whether multiple sessions of the intervention are beneficial and how the intervention translates to real life, such as delivering a speech *in vivo*. Participants predominantly had a subclinical level of PSA; so, the findings may not generalize to clinical SAD. Furthermore, therapeutic effects could be confounded by participant preference effects that are specific to the current self-guided VRET, namely the size and reaction of the audience, the number of speech prompts and the topic of the speech, and those that are general to intervention, such as autonomy (75) and attitude to intervention (76).

The manipulation of certain elements in the current VRET was successful in reducing anxiety. Going forward, machine learning could be used to identify the best candidate indicators of arousal, such as galvanic skin response (GSR), pupil diameter, heart rate (HR), and electromyography (77). Offering participants biofeedback about such arousal from heartrate and electroencephalography could enhance response to exposure therapy for SAD (78). Most studies (65%) offering biofeedback as an intervention for psychiatric disorders report symptom improvement (79), including control over threatening thoughts (80). Artificial intelligence could study the participant's voice stress patterns (29) and physiological arousal from virtual social threat and automatically up- or downgrade exposure to virtual threat (29). Further elements could also be added to enhance the realism of the virtual threat, for example, allowing avatars in the virtual audience to offer verbal auditory feedback (81) and allowing avatars to make natural small and gross movements, such as leaving the room or muttering to a neighbour (82).

This study is preliminary evidence of the feasibility of self-guided VRET. Self-guided VRET enables people with high PSA to voluntarily increase their exposure to virtual social threat, reduce short-term anxiety and physiological arousal, and improve perceived PSA up to a month after intervention. Such self-guided exposure could reduce the fear of negative evaluation, that is a core feature of social anxiety, and help people with high PSA to see the social threat objectively. Self-guided VRET has the potential to enhance engagement with services and augment treatment effects before, during and after treatment (31).

## Data Availability Statement

The datasets presented in this study can be found in online repositories. The names of the repository/repositories and accession number(s) can be found at: https://osf.io/tje8g.

## Ethics Statement

The studies involving human participants were reviewed and approved by Business, Law, and Social Sciences College Research Ethics Committee at Nottingham Trent University. The participants provided their written informed consent to participate in this study.

## Author Contributions

EZ, PP, NH, DB, and AS designed the study and VRET. SB developed the virtual interface of the virtual-reality exposure therapy. BH and RD conducted the data collection. PP, NH, EZ, BH, and RD performed the data analysis. PP wrote the first draft of the paper. All authors contributed to the interpretation of the results, writing and editing of the manuscript, and approved the final version of the manuscript.

## Conflict of Interest

The authors declare that the research was conducted in the absence of any commercial or financial relationships that could be construed as a potential conflict of interest.

## Publisher's Note

All claims expressed in this article are solely those of the authors and do not necessarily represent those of their affiliated organizations, or those of the publisher, the editors and the reviewers. Any product that may be evaluated in this article, or claim that may be made by its manufacturer, is not guaranteed or endorsed by the publisher.
